# Loss of CSF-contacting neuron sensory function is associated with a hyper-kyphosis of the spine reminiscent of Scheuermann’s disease

**DOI:** 10.1038/s41598-023-32536-1

**Published:** 2023-04-04

**Authors:** Laura Marie-Hardy, Lotfi Slimani, Giulia Messa, Zaineb El Bourakkadi, Annick Prigent, Celia Sayetta, Fanny Koëth, Hugues Pascal-Moussellard, Claire Wyart, Yasmine Cantaut-Belarif

**Affiliations:** 1 Orthopedic Surgery and Trauma Center, Pitié-Salpêtrière Teaching Hospital (AP-HP), 47 Boulevard de L’Hôpital, 75013 Paris, France; 2 Institut du Cerveau (ICM), Inserm U 1127, CNRS UMR 7225, Sorbonne Université (SU), 75013 Paris, France; 3 URP 2496 Laboratory Orofacial Pathologies, Imaging and Biotherapies, Dental School University Paris Cité, and Life Imaging Platform (PIV), Montrouge, France

**Keywords:** Morphogenesis, Developmental biology, Neuroscience, Medical research, Development

## Abstract

Scheuermann’s disease, also referred to as Scheuermann’s kyphosis, is the second most frequent spine deformity occurring in humans after adolescent idiopathic scoliosis (AIS), both with an unclear etiology. Recent genetic studies in zebrafish unraveled new mechanisms linked to AIS, highlighting the role of the Reissner fiber, an acellular polymer bathing in the cerebrospinal fluid (CSF) in close proximity with ciliated cells and mechanosensory neurons lining the central canal of the spinal cord (CSF-cNs). However, while the Reissner fiber and ciliary beating have been linked to AIS-like phenotypes in zebrafish, the relevance of the sensory functions of CSF-cNs for human spine disorders remains unknown. Here, we show that the thoracic hyper-kyphosis of the spine previously reported in adult *pkd2l1* mutant zebrafish, in which the mechanosensory function of CSF-cNs is likely defective, is restricted to the sagittal plane and is not associated with vertebral malformations. By applying orthopedic criteria to analyze the amplitude of the curvature at the apex of the kyphosis, the curve pattern, the sagittal balance and sex bias, we demonstrate that *pkd2l1* knock-outs develop a phenotype reminiscent of Scheuermann’s disease. Altogether our work consolidates the benefit of combining genetics and analysis of spine deformities in zebrafish to model idiopathic spine disorders in humans.

## Introduction

The spine is a segmented structure consisting of vertebrae intercalated with intervertebral discs that shares the same architecture and developmental origin in amniotes and bony fish^1,2^. In humans, spine disorders are characterized by defects of the vertebral alignment that can be classified according to their cause. While congenital deformities of the spine are observed at birth and involve malformations of the vertebral bodies contributing to a regionalized curvature of the spine^3^, syndromic deformities are acquired during life and are associated to diverse pathological states^4–6^. In contrast, idiopathic deformities of the spine are defined by atypical curves without underlying malformations that arise spontaneously from an unknown origin^7^.

The two most frequent idiopathic deformities of the spine occurring in humans are adolescent idiopathic scoliosis (AIS) and Scheuermann’s disease. Scheuermann’s disease affects 0.4–10% of the population^8,9^. It is a one-dimensional deformity occurring in the sagittal plane, associated with no vertebral malformations and consisting of a pronounced hyper-kyphosis of the thoracic spine that arises during the growth period in otherwise healthy patients. Its etiology remains unclear but growth irregularities and multiple genetic factors are reported to be involved in this disease^9,10^. AIS is the most frequent form of idiopathic spine disorders affecting almost 4% of the population^1,7^ and is defined by a three-dimensional misalignment of the vertebrae occurring during the period of growth.

While the pathogenesis of AIS is still a subject of debate in humans, zebrafish allowed for substantial advances in the identification of novel underlying mechanisms. They involve the cerebrospinal fluid (CSF), a protein-rich solution filling the cavities of the nervous system whose constant circulation is generated by the coordinated beating of motile cilia projecting from the walls of the brain and spinal cord cavities^11–13^. The CSF contains the Reissner fiber, an acellular thread conserved in many vertebrates that runs from the roof of the third ventricle down to the caudal end of the central canal and that is formed by the cilia-dependent aggregation of the SCO-spondin glycoprotein^14,15^. The reduction of cilia motility in *c21orf59* mutants leads in juvenile zebrafish to three-dimensional spine curves recapitulating the features of AIS^16^. Remarkably, zebrafish mutants devoid of the Reissner fiber develop similar spine curves characterized by an increased misalignment of the vertebrae both in the sagittal and coronal planes^17–19^. This body of work revealed that defects in the assembly of the Reissner fiber is a key mechanism driving spine deformity across different genetic models of AIS.

In the spinal cord, the Reissner fiber is confined in the central canal where it forms a sensory system with CSF-contacting neurons (CSF-cNs), a population of interoceptive neurons projecting their apical extension into the CSF^20^. To detect spine curvature, CSF-cNs require the transient receptor potential channel Trpp2 (formerly Trpp3) or Pkd2l1^21^ enriched in their apical extension^22^ and the Reissner fiber^23^. *pkd2l1* zebrafish mutants in which the mechanosensory function of CSF-cNs is abolished in the larva^21^ develop at the adult stage an increased curvature of the spine that occurs in the sagittal plane and increases in severity between 12 and 19 months of age^22^. Thus, Pkd2l1 is required both for the mechanosensory function of CSF-cNs and in the maintenance of spine alignment throughout life.

This body of work highlights the influence of the cellular niche formed around the Reissner fiber, together with ciliated cells driving CSF flow and CSF-cNs, on spine alignment. However, while the Reissner fiber and ciliary beating have been directly linked to AIS-like phenotypes in zebrafish, the relevance of the mechanosensory function of CSF-cNs for spine deformities in humans remains unknown. Here, we show that the pronounced curvature of the spine reported in *pkd2l1* zebrafish mutants is not associated with vertebral malformations but is idiopathic and restrained to the sagittal plane. By applying orthopedic criteria to analyze spine misalignments in *pkd2l1* mutants, including the amplitude and location of hyper-kyphosis in the thoracic spine, curve pattern, sagittal balance, and sex bias, we further confirm that the loss of the mechanosensory function of CSF-cNs in *pkd2l1* mutants is associated with a deformity reminiscent of Scheuermann’s disease.

## Results

### Spine hyper-kyphosis in *pkd2l1* mutants is not associated with vertebral malformations

**Figure 1:**
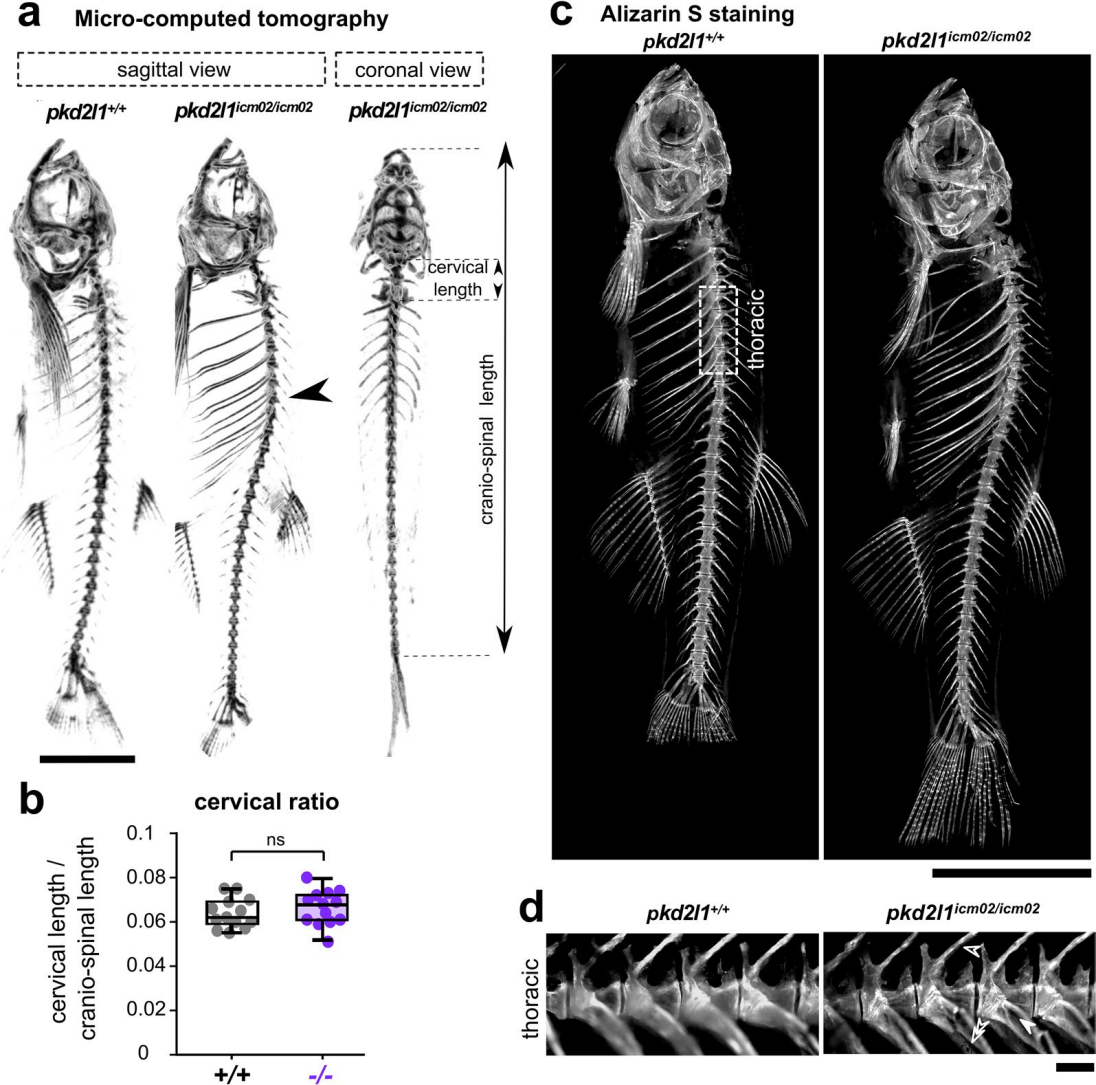
*pkd2l1* null mutants develop during adulthood an idiopathic hyper-kyphosis of the thoracic spine. (**a**) 3D micro-computed tomography reconstruction images of a 18 months-old wild-type (*pkd2l1*^+*/*+^, left sagittal view) and a *pkd2l1*^*icm02/icm02*^ mutant sibling (middle, sagittal view; right, frontal view). *pkd2l1*^*icm2/icm02*^ mutants exhibit a thoracic hyper-kyphosis of the spine (arrowhead) but show no deformity in the coronal plane. Scale bar: 0.5 cm. (**b**) Distribution at 18 months-old of the cervical ratio, defined as the cervical length divided by the cranio-spinal length, in wild-type (grey, + / + , n = 13) and *pkd2l1*^*icm02/icm02*^ mutants (purple, −/−, n = 13). Each point represents a single animal. Boxplots represent median values ± IQR. ns: *p* > 0.05, Mann–Whitney test. (**c**) Sagittal views of Alizarin S stained-skeletal preparations of 18 months-old wild-type (*pkd2l1*^+*/*+^, left) and a *pkd2l1*^*icm02/icm02*^ mutant sibling (right). Scale bar: 0.5 cm. (**d**) High-magnification fluorescence images of Alizarin S in the thoracic region of the spine (as defined in (**c**), dotted line rectangle) in a wild-type (left) and a *pkd2l1*^*icm02/icm02*^ sibling (right). Neural arches (empty arrowhead), hemal arches (double empty arrowhead) and ribs (solid arrowhead) are highlighted for the *pkd2l1*^*icm02/icm02*^ mutant. Note that in both genotypes, no vertebral dislocation, malformation or fusion are observed. Scale bar: 500 µm.

The morphoanatomy of the zebrafish spine, as in humans, is characterized by a natural longitudinal curvature at the thoracic level, referred to as kyphosis^1^. *pkd2l1*^*icm02/icm02*^ mutants develop an accentuated curvature of the thoracic spine^22^. To clarify whether hyper-kyphosis in *pkd2l1* mutants is idiopathic, we combined micro-computed tomography and Alizarin-S staining on skeletal preparations of 18 months old adult zebrafish (Fig. 1), a stage at which the deformities of the thoracic spine are fully developed^22^. First, we examined whether spine curvatures are restricted to the sagittal plane (Fig. 1a). We observed that *pkd2l1* knock-outs (13 out of 13) exhibited no deviation in the coronal plane similarly to their wild-type siblings, confirming that they do not develop three-dimensional misalignments of the spine.

Several zebrafish mutant models of congenital syndromes recapitulate pronounced curvatures of the thoracic spine^24–26^. Thus, we investigated whether the phenotype of adult *pkd2l1*^*icm02/icm02*^ mutants could arise from vertebral malformations. In human, complex syndromic hyper-kyphosis, such as in Klippel-Feil syndrome, is associated with a reduced cervical length^27^. Given that the length of the cervical segment can vary with the body size of the animals, we used the cervical ratio defined as the length of the cranio-cervical segment normalized by the total axial length (Fig. 1a). Figure 1b shows that *pkd2l1*^*icm02/icm02*^ mutants exhibited cervical ratios similar to that of wild-type siblings (*p* = 0.44; Mann–Whitney test). Moreover, the number of thoracic vertebrae ranged between 8.1 ± 0.1 and 8.6 ± 0.2 in wild-type and *pkd2l1*^*icm02/icm02*^ mutants, respectively (mean ± SEM; n = 13 and 13 wild-type and mutant animals respectively; *p* = 0.07; Mann–Whitney test). This is in accordance with the axial formula previously reported for rib-bearing vertebrae observed in the adult zebrafish spine^28^ and suggests that the phenotype of *pkd2l1*^*icm02/icm02*^ mutants does not correlate with spine patterning defects.

Next, we analyzed the morphoanatomy of the spine in *pkd2l1*^*icm02/icm02*^ mutants using Alizarin-S staining. Similar intensities of Alizarin-S were observed in wild-type and mutant siblings (Fig. 1c), suggesting that no obvious mineralization defects occur in *pkd2l1*^*icm02/icm02*^ mutants. By examining the morphoanatomy of the vertebrae along the spine (Fig. 1c), we found no vertebral hypoplasia, fusion, dislocation or butterfly-like vertebral bodies in mutant backgrounds (13 out of 13), as previously reported in zebrafish genetic models of congenital disorders affecting the spine shape^29–31^. Instead, we observed that vertebrae retained a normal structure in *pkd2l1*^*icm02/icm02*^ mutants (13 out of 13) even at the site of curvature (thoracic, Fig. 1d) where differentiated centra, neural and hemal arches as well as ribs projecting from the thoracic vertebrae developed. Thus, adult *pkd2l1* zebrafish mutants retain a normal architecture and morphoanatomy of the spine. Altogether, these results show that the hyper-kyphotic phenotype observed in *pkd2l1* mutants at adult stage is not associated with vertebral malformations and is rather idiopathic.

### *pkd2l1* mutant larvae exhibit an intact Reissner fiber

**Figure 2:**
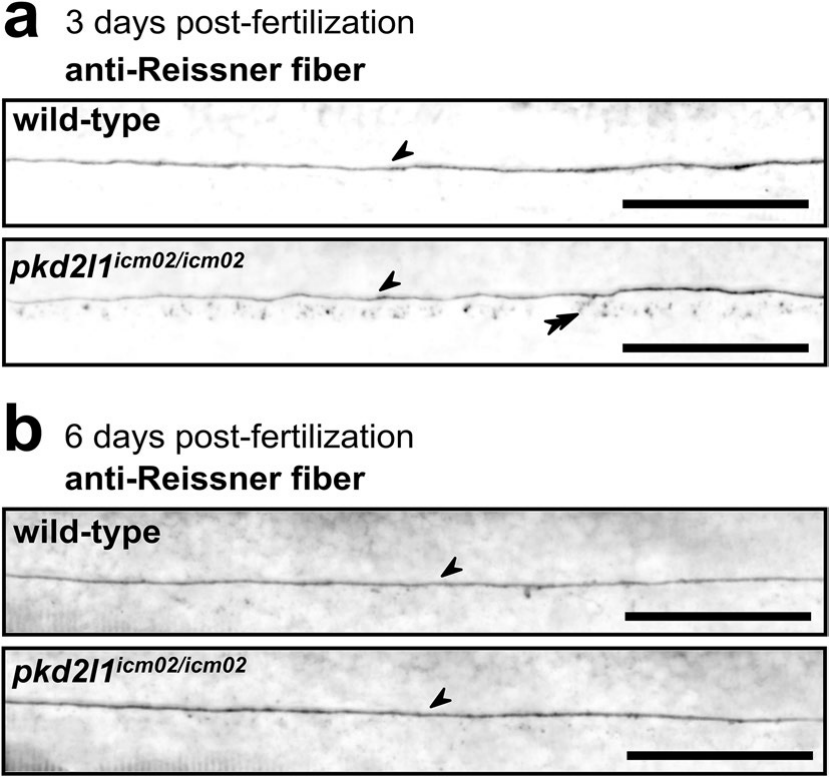
*pkd2l1* mutant larvae retain a normally assembled Reissner fiber in the central canal of the spinal cord. Sagittal views of representative immunostainings against the Reissner fiber material imaged in the central canal of the spinal cord of 3 dpf (**a**) and 6 dpf (**b**) wild-type (top, one representative larva out of 8/9 at 3/6 dpf, respectively), and *pkd2l1*^*icm02/icm02*^ mutants (bottom, one representative larva out of 7/9 at 3/6 dpf, respectively). Arrows denote the presence of a continuous Reissner fiber in the central canal of the spinal cord. Double arrowheads indicate the presence of Reissner fiber-positive material in the floor plate at 3 dpf. Larvae are oriented rostral to the left and dorsal to the top. Scale bars: 50 µm.

In zebrafish, spine misalignments reminiscent of AIS are driven by dysfunctions in the pathway involving CSF-cNs and the Reissner fiber. The presence of an intact Reissner fiber is required for the development of a straight body axis in the embryo^15^. Its disassembly is associated with the onset of scoliosis^17,18^ and with a downregulation, at embryonic and post-embryonic stages, of the expression level of the Urp1 and Urp2 neuropeptides^18,19,32^ that are expressed in CSF-cNs^33^. Embryonic *pkd2l1* mutants have a straight body axis and retain accordingly a normal Reissner fiber^32^. The loss of the mechanosensory activation of CSF-cNs upon trunk bending was reported in *pkd2l1* mutant larvae^21^ at 6 days post-fertilization larvae. We tested whether larval *pkd2l1* mutants retained a normally assembled Reissner fiber (Fig. 2). We used the L1P1b antiserum to stain against the Reissner fiber in the central canal of the spinal cord at 3 and 6 dpf. Figure 2 shows that *pkd2l1*^*icm02/icm02*^ mutant larvae retained an intact Reissner fiber that was undistinguishable from that of wild-type siblings at 3 dpf (Fig. 2a) and 6 dpf (Fig. 2b). These results confirm that the Reissner fiber is not disrupted in *pkd2l1* mutant larvae, at earlier time-points than the stage at which hyper-kyphosis develops.

### The orthopedic characterization of spine hyper-kyphosis in *pkd2l1* mutants corroborates a similarity to Scheuermann’s disease

**Figure 3:**
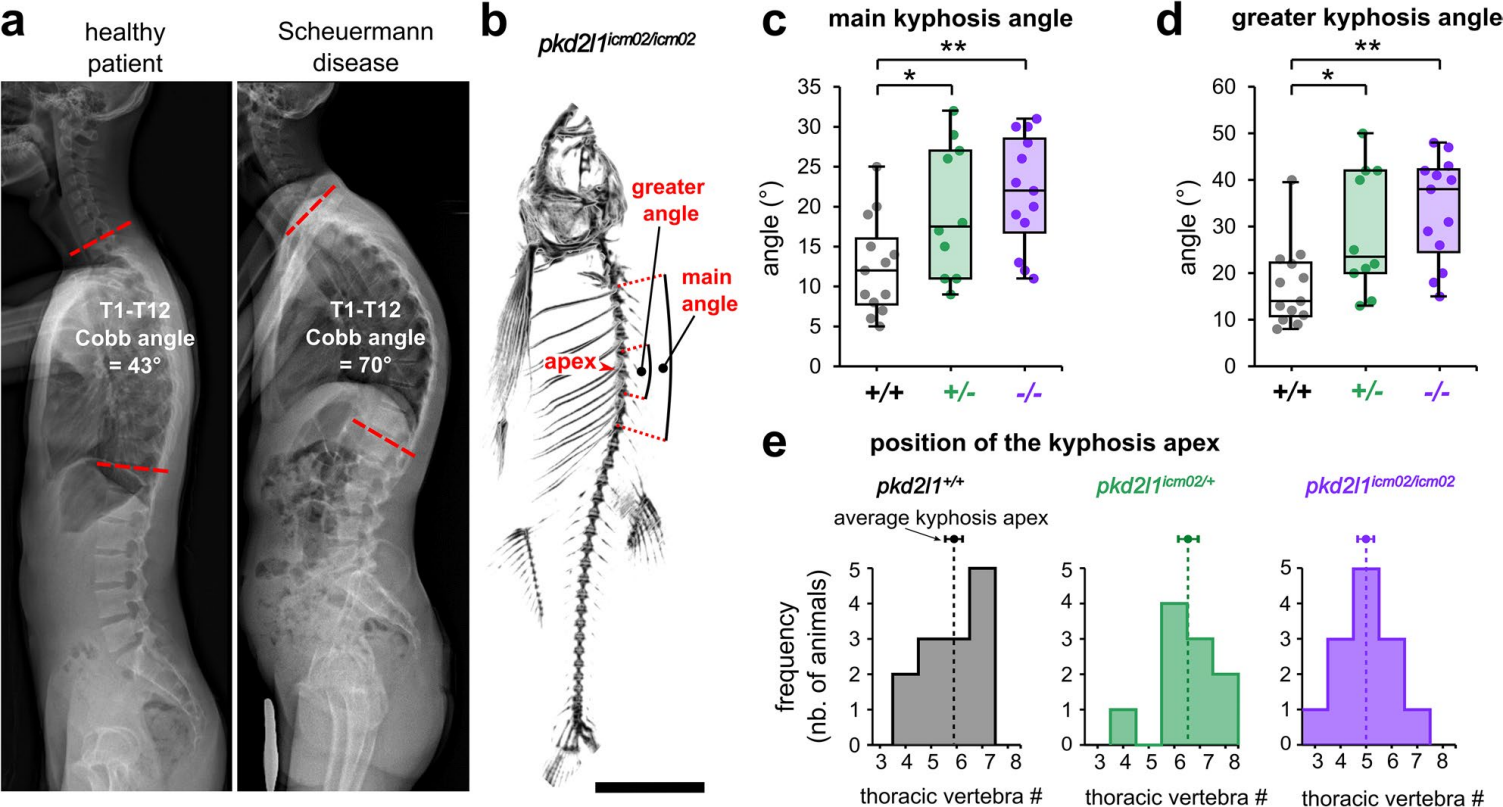
Pkd2l1 loss-of-function induces a hyper-kyphosis of the thoracic spine reminiscent of Scheuermann’s disease. (**a**) X-ray radiographs in the sagittal plane of a healthy patient (left; male, 20 years old) and of a patient with Scheuermann’s idiopathic disease (right; male, 20 years old). The deformity of the thoracic spine (right) is characterized by a Cobb angle between the first (T1) and the twelfth (T12) thoracic vertebra of 70° and is associated with no vertebral malformations. (**b**) Representative 3D micro-computed tomography reconstructions of a *pkd2l1*^*icm02/icm02*^ mutant (18 months-old) used to exemplify the evaluation in the sagittal plane of the main kyphosis angle, the greater kyphosis angle and the kyphosis apex. Scale bar: 0.5 cm. (**c**, **d**) Distribution of the main kyphosis angle (**c**) and greater kyphosis angle (**d**) in wild-type (black, + / + , n = 13), heterozygous (green, + /−, n = 10) and *pkd2l1*^*icm02/icm02*^ mutants (purple, −/−, n = 13). Boxplots represent median values ± IQR. Each point represents a single animal. *: *p* < 0.05, **: *p* < 0.01, Mann–Whitney test. (**e**) Distribution of the position of the apex of the kyphotic curve. The average position of the kyphosis apex is represented for each genotype (arrow, mean ± SEM).

**Figure 4:**
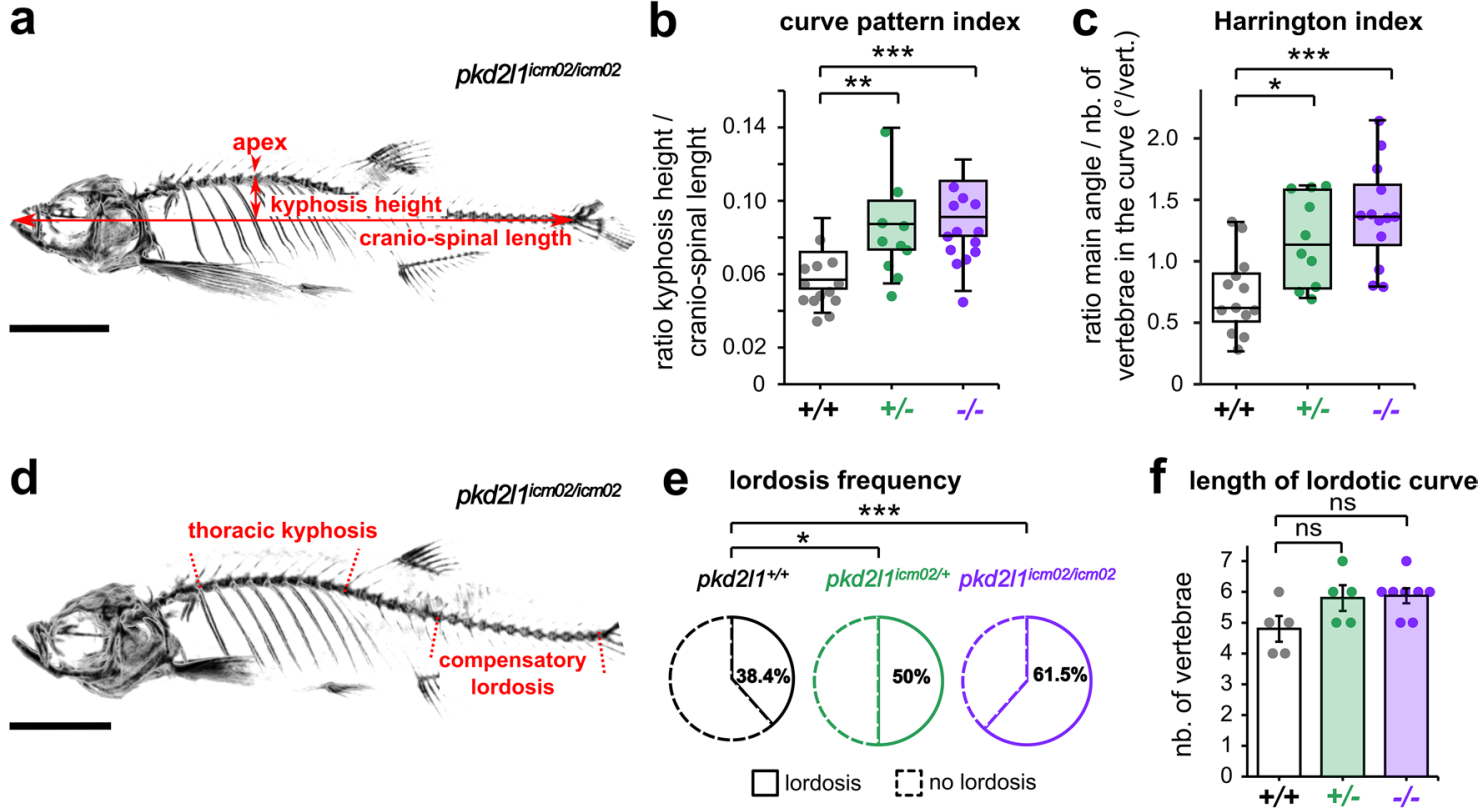
Adult *pkd2l1* mutants develop a sagittal imbalance of the spine. (**a**) Representative 3D micro-computed tomography reconstruction of a *pkd2l1*^*icm02/icm02*^ mutant (18 months-old) used to exemplify the evaluation in the sagittal plane of the kyphosis height, the cranio-spinal length and the kyphosis apex. Scale bar 0.5 cm. (**b, c**) Distribution of the curve pattern index (**b**) and Harrington index (**c**) in wild-type (black, + / + , n = 13), heterozygous (green, + /−, n = 10) and *pkd2l1*^*icm02/icm02*^ mutants (purple, −/−, n = 13). Boxplots represent median values ± IQR. Each point represents a single animal. *: *p* < 0.05, **: *p* < 0.01, ***: *p* < 0.001, Mann–Whitney test. (**d**) Representative 3D micro-computed tomography reconstruction used to identify in the sagittal plane the thoracic kyphosis and a compensatory lordosis in the caudal region of the spine, exemplified in a 18 months-old *pkd2l1*^*icm02/icm02*^ mutant. Scale bar: 0.5 cm. (**e**) Pie charts representing the frequency of caudal lordotic curves in wild-type (black, n = 13), heterozygous (green, n = 10) and *pkd2l1*^*icm02/icm02*^ mutants (purple, n = 13). *: *p* < 0.05, ***: *p* < 0.001, Chi-Square test. (**f**) Histogram representing the length of the compensatory curve in lordotic wild-type (grey, + / + , n = 5), heterozygous (green, + /−, n = 5) and *pkd2l1*^*icm02/icm02*^ animals (purple, −/−, n = 6). Each point represents a single animal. Histograms represent mean values ± SEM. ns: *p* > 0.05, Mann–Whitney test.

**Figure 5:**
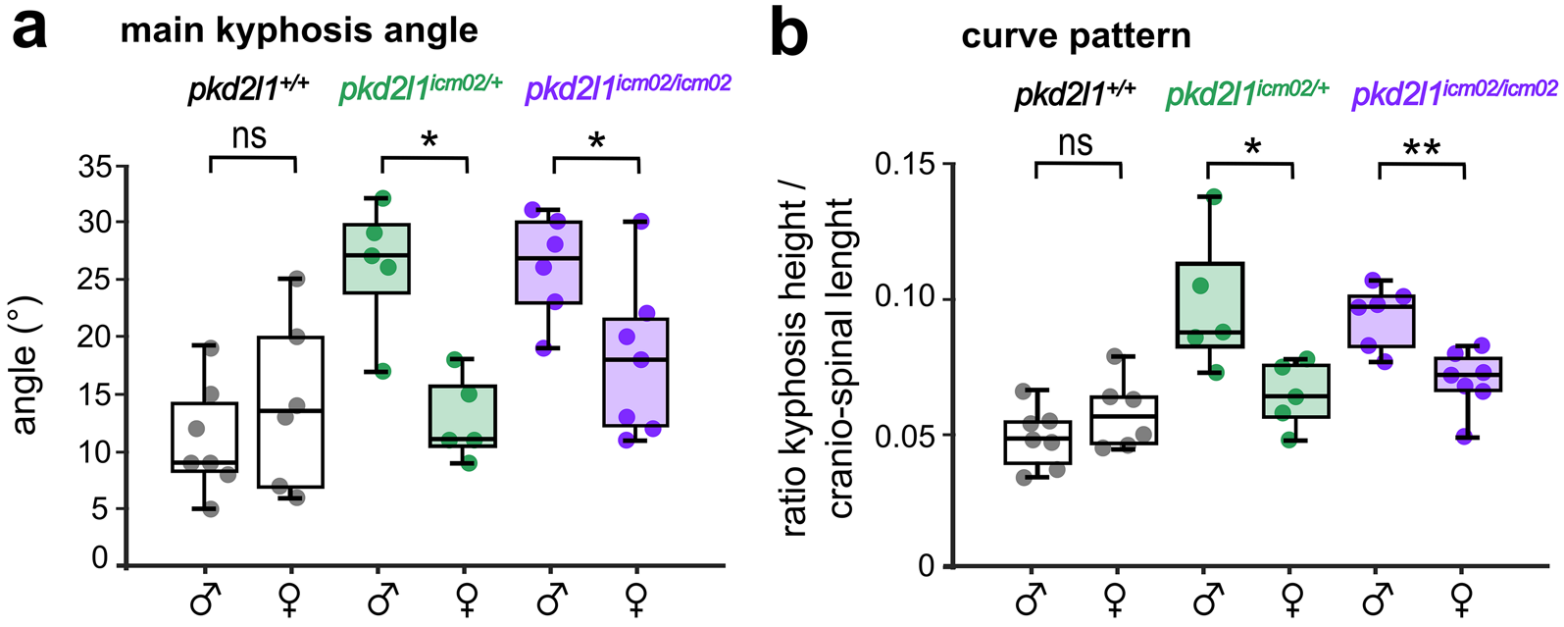
The severity of spine hyper-kyphosis in *pkd2l1* mutants is sexually biased towards males. Distribution of the main kyphosis angle (**a**) and the curve pattern (**b**) in 18 months-old males and females of wild-type (black, + / + , n = 7 and 6 males and females respectively), heterozygous (green, + /, n = 5 and 5 males and females respectively) and mutant genotypes (purple, −/−, n = 6 and 7 males and females respectively). Each point represents a single animal. Boxplots represent median values ± IQR. *: *p* < 0.05, **: *p* < 0.01, Mann–Whitney test.

To test whether the phenotype of *pkd2l1* mutants is reminiscent of Scheuermann’s disease, we used micro-computed tomography and performed an orthopedic analysis of the zebrafish spine based on the criteria allowing to define this pathology as such during the diagnosis process in patients. We investigated three features of Scheuermann’s kyphosis in *pkd2l1* mutants: the amplitude and location of hyper-kyphotic curves (Fig. 3), the curve pattern and the sagittal balance of the spine (Fig. 4), and sex bias (Fig. 5).

In humans, Scheuermann’s disease is identified by the accentuation of the natural kyphosis of the thoracic spine and is characterized by a T1-T12 Cobb angle ranging between 50° and 70° and higher for severe cases^9,34^ (exemplified in Fig. 3a). Upon radiographic examination on the sagittal plane, a Cobb angle higher than 50° distributed on at least three consecutive thoracic vertebrae without associated malformations is the major criterion for patient diagnosis^9,10,34,35^. Thus, we analyzed the Cobb angle between the first and the last thoracic vertebra, referred to as main angle, and the greater kyphosis angle defined as the Cobb measure between three to four adjacent vertebrae surrounding the apex of the curve (Fig. 3b). We observed that the median values of the main and greater kyphosis angles show a significant 1.75 and 2.7- fold increase, respectively, in *pkd2l1* homozygous mutants compared to their wild-type siblings (Fig. 3c, d; *p* = 0.004 and 0.001, respectively; Mann–Whitney test). This was accompanied by a slight but non-significant cranialization of the kyphotic curve in *pkd2l1*^*icm02/icm02*^ mutants (Fig. 3e) where the average kyphosis apex was positioned at T5 ± 0.3 compared to wild-type siblings where the average apex was at T5.8 ± 0.3 (mean ± SEM; n = 13 animals; *p* = 0.07; Mann–Whitney test). Interestingly, heterozygous *pkd2l1*^*icm02/*+^ animals showed an intermediate phenotype characterized by a 1.45- and 1.95- fold increase of the main and greater kyphosis angles, respectively, compared to wild-type siblings (Fig. 3c, d; *p* = 0.04 and 0.02, respectively; Mann–Whitney test). Overall, *pkd2l1* zebrafish mutants exhibited a typical 1.5- to 2.3- fold increase of the thoracic Cobb angle, similar to the ratios reported in patients with Scheuermann’s disease^9,36,37^.

We next examined whether *pkd2l1* zebrafish mutants phenocopy the sagittal imbalance reported in patients with Scheuermann’s disease^36,37^. The orthopedic evaluation of curve patterns in patients relies on sagittal parameters taking into account measures in reference to the pelvis such as the pelvic incidence, the sacral slope and the pelvic tilt^38,39^. The absence of limbs and pelvis in zebrafish precludes the assessment of sagittal alignment according to the orthopedic standards in humans. Thus, we evaluated the degree of deviation of the kyphotic curve relative to the entire spine by quantifying the curve pattern index, defined as the ratio between the kyphosis height and the cranio-spinal length^40^ (Fig. 4a, b). We observed that its median value showed a significant 1.6- fold increase in homozygous *pkd2l1* mutants and a 1.5- fold increase in heterozygous *pkd2l1* animals compared to wild-type siblings (Fig. 4b; *p* = 4.8 E-04 and 0.003, respectively; Mann–Whitney test). We also evaluated the distribution of the kyphotic curve along the spine by measuring the Harrington index, commonly used to follow curve progression in patients^41,42^. We observed a significant 2.2- fold and 1.8- fold increase of the median Harrington index in *pkd2l1*^*icm02/icm02*^ and *pkd2l1*^*icm02/*+^ animals compared to wild-type siblings, respectively (Fig. 4c; *p* = 4.1 E-04 and 0.01, respectively; Mann–Whitney test). Sagittal imbalance in patients with Scheuermann’s disease includes the appearance of lumbar lordosis^36^ to compensate for thoracic hyper-kyphosis and maintain a stable center of gravity as well as postural control^38,39,43^. Thus, we quantified the frequency at which lordosis occurs in the caudal spine (Fig. 4d, e). We observed that *pkd2l1*^*icm02/icm02*^ mutants, and to a lesser extent *pkd2l1*^*icm02/*+^ animals, showed a significantly higher lordosis frequency (Fig. 4e; *p* = 2 E-06 and 0.01, respectively; Chi-Square test). However, we did not observe significant differences in the length of the caudal compensatory curves amongst lordotic *pkd2l1*^*icm02/icm02*^ and *pkd2l1*^*icm02/*+^ animals compared to wild-type siblings (Fig. 4f; *p* = 0.06 and 0.2, respectively; Mann–Whitney test). Taken together, our results show that the phenotype of Pkd2l1-defective mutants evokes the sagittal imbalance observed in patients with Scheuermann’s disease.

Spine hyper-kyphosis in Scheuermann’s disease patients is more severe in men than in women^44–48^. Thus, we investigated whether the phenotype of *pkd2l1* mutants is gender gender-biased. We compared the distribution of the main kyphosis angle (Fig. 5a) and the curve pattern index (Fig. 5b) between males and females amongst wild-type, heterozygous and *pkd2l1*^*icm02/icm02*^ groups. Our results show that the main kyphosis angle and curve pattern index are comparable between wild-type males and females (*p* = 0.5 and 0.4, respectively; Mann–Whitney test). In contrast, *pkd2l1*^*icm02/icm02*^ males exhibit a 1.5- fold higher median kyphosis angle and a 1.3- fold higher median curve pattern index compared to females of the same genotype (*p* = 0.03 and 0.004, respectively; Mann–Whitney test). Similarly, *pkd2l1*^*icm02/*+^ males exhibit a 2.5- fold higher median kyphosis angle and a 1.4- fold higher median curve pattern index compared to females of the same genotype (*p* = 0.01 and 0.03, respectively; Mann–Whitney test). Our results show that the severity of hyper-kyphosis is sexually biased towards males in *pkd2l1*^*icm02/icm02*^ mutants. Altogether, our results confirm that Pkd2l1-defective mutants develop a hyper-kyphosis of the thoracic spine that is exaggerated for males and therefore reminiscent of Scheuermann’s disease.

## Discussion

Here, we show that the hyper-kyphosis of the thoracic spine observed in adult *pkd2l1* mutants is restricted to the sagittal plane and is not associated with vertebral malformations. By employing an orthopedic-based approach on micro-computed tomography data, we show that Pkd2l1-defective animals, previously shown to lack CSF-cN mechanosensory response at larval stage^21^, exhibit a round hyper-kyphotic curve at the thoracic level that is associated with a sagittal imbalance involving a caudal lordosis of the spine. We also report that hyper-kyphosis in mutants is more severe in males. Based on these observations, we propose that the closest human pathology is Scheuermann’s disease and that the *pkd2l1* knock-out is a relevant model for this idiopathic spine deformity.

Our results also show that adult heterozygous *pkd2l1* animals develop a hyper-kyphotic phenotype characterized by increased kyphosis angles, curve pattern and Harrington indexes compared to wild-type siblings, but of intermediate severity compared to homozygous mutants of the same age. This indicates a dose-dependent effect of the Pkd2l1 channel on the alignment of the spine, suggesting that a controlled amount of Pkd2l1 protein addressed at the apical extension of CSF-cNs may be required to sustain their mechanosensory function. This working model is supported by evidence that the spontaneous activity of CSF-cNs is reduced in heterozygous *pkd2l1*^*icm02/+*^ embryos and nearly abolished in homozygous mutant siblings^22^.

Zebrafish are considered adult when they reach sexual maturity around 3 months of age and are subject to spontaneous spine deformities with elderliness. The severity of the curvatures increases linearly in animals over 20 months of age^49,50^ and is accompanied by degenerative changes in vertebral structures^50^. Here, the orthopedic characterization of spine hyper-kyphosis in *pkd2l1* mutants relies on comparisons with wild-type siblings of the same age, limiting the bias related to the spontaneous occurrence of spine misalignments in mutants. Moreover, we report that no vertebral dislocations nor obvious changes in bone mineralization, assessed qualitatively via Alizarin-S staining, occur in *pkd2l1* knock-outs compared to their wild-type siblings, making unlikely that mutant animals develop spine misalignments of degenerative origin. Hyper-kyphosis in *pkd2l1* mutants has originally been reported over three independent generations^22^ and observed as early as 12 months of age. However, Scheuermann’s disease is diagnosed around the pubertal period and in young adults, making the onset of hyper-kyphosis in zebrafish *pkd2l1* mutants distinct from that characterizing the human pathology. This discrepancy might be linked to the fact that unlike humans, zebrafish grow throughout their life span^49^ and suggests that compensatory mechanisms might occur to sustain a normal alignment of the spine in *pkd2l1* mutants prior to adult stage. Future investigations will be needed to decipher whether targeting CSF-cNs specifically during the juvenile period of growth is sufficient to drive similar spine alignment defects.

Our study shows that the lack of mechanosensation in CSF-cNs at larval stage correlates with spine hyper-kyphosis reminiscent of Scheuermann’s disease in adults. In zebrafish, CSF-cNs are also involved in spine deformities reminiscent of AIS, which rely on the maintenance of a controlled expression level of Urp1 and Urp2, two neuropeptides expressed from the embryonic life to adulthood in these sensory neurons^33^. Indeed, *urp1;urp2* double mutants were recently reported to develop AIS-like phenotypes is zebrafish^51,52^. Moreover, a downregulation of Urp1/2 peptides expression level was reported in AIS mutant models with defective Reissner fiber acting upstream of CSF-cNs^18,19^. In the embryo, *pkd2l1* mutants retain a normally assembled Reissner fiber and express *urp1* and *urp2* at similar levels compared to wild-type siblings^32^ suggesting that the mechanosensory function of CSF-cNs does not control Urp1/2 expression level. Our work further supports that mechanosensation and neuropeptide expression, both taking place in CSF-cNs, are two mechanisms that influence differentially the alignment of the spine, which may explain why *pkd2l1* mutants develop spine deformities distinct from AIS-like phenotypes.

The main hypothesis underlying the etiology of Scheuermann’s disease is genetic. Indeed, a higher occurrence of this deformity has been reported in monozygotic twins, indicating a genetic contribution to the etiology of the disease^46^. Families with an autosomal dominant inheritance pattern and a high penetrance are also documented^57^. Mutations in *COL2A1* and *COL9A3* encoding for collagens enriched in intervertebral disks^58^ were recently reported in patients with Scheuermann’s kyphosis and associated with a defective growth of the cartilage endplates at the vertebrae margins^59,60^. Interestingly, recent findings report that murine intervertebral disks are enriched with UTS2R^61^, a receptor for Urp1/2 peptides expressed by CSF-cNs, which is asymmetrically expressed in the paravertebral muscles of AIS patients^62^. This raises the question of whether CSF-cNs could influence the development of intervertebral discs or muscles surrounding the vertebrae during growth.

Our work links zebrafish mutants in which mechanosensation is defective in CSF-cNs with Scheuermann’s disease. But what could be the underlying mechanisms? In the zebrafish larva, the Reissner fiber is required for the asymmetric mechanosensory activation of CSF-cNs during tail bending^23^. Two hypotheses have been proposed to explain the selective activation of the sensory neurons located on the concave side of the curvature: either a direct contact between the Reissner fiber and CSF-cNs or local enhancements of CSF flow near the apical extension of the cells^23^. Interestingly, MRI studies in Scheuermann’s disease patients reported frequent epidural lipomatosis and syringomyelia^53,54^ that are well known to impair CSF pressure and flow. Moreover, a dedicated study shows that 41% of Scheuermann’s disease patients recapitulate the typical features of spinal epidural lipomatosis^55^, known to produce increased epidural pressure and consecutively increase CSF pressure^56^. This suggests a possible correlation between CSF circulation and Scheuermann’s disease.

By characterizing the relevance of zebrafish mutants defective in the mechanosensory function of CSF-cNs for Scheuermann’s disease, our work highlights the influence of the niche formed around the Reissner fiber, known to be essential for spine morphogenesis. Dysfunctions in the Reissner fiber or in CSF-cN’s mechanosensory functions lead to spine misalignments reminiscent of AIS and hyper-kyphosis, respectively, suggesting a common origin linked to this axial sensory system for eliciting distinct idiopathic spine disorders.

## Methods

### Animal husbandry

Animals were generated from incrosses of *pkd2l1*^*icm02/*+^ parents resulting in a progeny (one generation) of wild-type (25%), heterozygous (50%) and mutant siblings (25%). Animals were maintained in a 14 / 10 h light cycle at 28 °C until 6 days post-fertilization and then raised in 20L aquarium at 25 °C until 18 months-old. Animal handling and protocols were carried out in accordance with the European Communities Council Directives (2010/63/EY) and French law (87/848) and approved by the Paris Brain Institute (Institut du Cerveau). An approval agreement for experimentation on adult animals was obtained from the ethic committee of the French Ministry for research (APAFIS agreement 2018071217081175).

### X-Ray radiographs involving human patients

Full spine X-ray radiographs were obtained as part of patients’ care course (non-interventional) in accordance with the Declaration of Helsinki of the World Medical Association revised in 2013 for medical research involving human subjects. An approval agreement for the collection and publication of X-ray radiographs from human patients was obtained from the ethic and data protection committee of the Assistance Publique des Hôpitaux de Paris (APHP agreement 20220916143818).

### Genotyping

Genotyping was performed from genomic DNA isolated from fin clips (for adults) or whole larvae (Fig. 2) by overnight Proteinase K treatment. The *pkd2l1*^*icm02*^ mutation was genotyped by PCR at 61 °C using forward (TGTGTGCTAGGACTGTGGGG) and reverse (AGGGCAAGAGAATGGCAAGACG) primers to generate a 400 bp product. Wild-type sequences were cleaved by *Sac1* to produce a 350 bp and a 50 bp band, while the mutant sequences were resistant to digestion^21^.

### Micro-computed tomography and scannographic analysis

Animals were anesthetized using 0.02% Tricain (MS-222, SIGMA) and transferred into imaging chambers filled with Tricain-containing fish water in order to avoid positioning artifacts due to a contact with a dense surface^40^. Acquisitions were performed on a Quantum FX Caliper (PERKIN ELMER, CALIPER LIFE SCIENCES, Hopkinton, MA, USA) with a 59 µm spatial resolution using a 30 mm field of view. Imaging required up to 3 min per animal. Rigaku software (RIGAKU, Tokyo, Japan) was used for real time 3D reconstructions and RadiAnt DICOM Viewer (MEDIXANT, Poznan, Poland) for image processing. The quantitative analysis of spine alignment was performed on 3D reconstructions oriented in the sagittal plane except for Fig. 1b (coronal plane).

The cranio-cervical length was calculated from the tip of the jaw to the caudal-most vertebra of the spine, excluding the tail fin set (Fig. 1a, b). The cervical length was defined as the segment extending from the cranio-cervical junction—excluding the Weberian apparatus—to the anterior endplate of the first thoracic vertebra (Fig. 1a, b). The cervical ratio was defined as the ratio between the cervical length and the cranio-spinal length (Fig. 1b). We defined the main kyphosis angle as the Cobb measure between the anterior endplate of the first thoracic vertebra and the posterior endplate of the last thoracic vertebra (Fig. 3b, c). The greater kyphosis angle was defined as the Cobb measure between three to four adjacent vertebrae surrounding the apex of the thoracic curve (Fig. 3b, d). The apex of the thoracic curve was defined as the most distant thoracic vertebra from the cranio-cervical axis (Figs. 3b, e, 4a). The kyphosis height was defined as the length of the line segment starting at the apex of the kyphotic curve and dropped down perpendicularly to the sagittal axis (Fig. 4a). The curve pattern index was defined as the ratio between the kyphosis height and the cranio-spinal length (Fig. 4b). The Harrington index was calculated as the ratio between the main kyphosis angle and the number of vertebrae composing the thoracic curve (Fig. 4c). Caudal curvatures were considered lordotic when the line segment joining the center of the caudal vertebral bodies exhibited an angular deviation higher than 9° on at least 4 consecutive vertebrae (Fig. 4d, e, f).

### Skeletal preparations, Alizarin S staining and imaging

Animals were euthanized in 0.2% Tricain (MS-222, SIGMA) and fixed in 4% paraformaldehyde (PFA, 15714, EUROMEDEX). After evisceration, samples were depigmented in a solution containing 1% KOH and 3% H_2_O_2_ for 24 h, washed 3 times during 10 min in tap water and incubated in 30% borax for 24 h. Soft tissues were then digested in a solution containing 2% borax and 0.5% Trypsin (T4799, SIGMA) over one day and one night. Skeletal tissues were stained using a solution containing 1 mg/mL Alizarin S (A5533, SIGMA) and 1% KOH. Samples were washed 3 times during 10 min in tap water and incubated in a 2% borax solution over two nights. Samples were then stored in glycerol until imaging. Stained skeletal preparations (Fig. 1c, d) were imaged on a AZ100M macroscope (NIKON) using a 532–554 nm excitation/573–613 nm emission filter. The same exposure times were used to image mutant and wild-type siblings (Fig. 1c, d).

### Immunohistochemistry

Immunohistochemistry was performed as described in^15^. 3 and 6 days post-fertilization larvae were euthanized in 0.2% Tricain (MS-222, SIGMA), fixed 2 h in 4% PFA, 3% sucrose at 4 °C, and washed 3 times during 10 min in 1X PBS. Skin from the rostral part of the trunk was removed and samples were blocked overnight in 0.7% Triton X-100, 1% DMSO, 10% NGS and 2 mg/mL BSA. Larvae were then incubated overnight at 4 °C in a solution containing 0.5% Triton X-100, 1% DMSO, 1% NGS, 1 mg/mL BSA and L1P1b anti-serum^63^ diluted at 1:200. Alexa Fluor Plus 488 goat anti-Rabbit IgG (H + L) secondary antibodies (A32731, INVITROGEN) were diluted at 1:500 in a solution containing 0.7% Triton X-100, 1% DMSO, 10% NGS and 2 mg/mL BSA and incubated 2.5 h at room temperature. Larvae were mounted laterally in IBIDI Antifade Mounting medium and imaged on a confocal spinning disk microscope (INTELLIGENT IMAGING SYSTEMS, Denver) equipped with a 20X water immersion objective. Samples were subsequently unmounted and subjected to genomic DNA extraction and genotyping. Images were processed using Fiji^64^. Maximal Z-projections of 10 microns in depth are represented in Fig. 2.

### Statistical analysis

Values in Figs. 1b, 3c, d 4b, c, 5a, b are represented as boxplots (median ± interquartile range, *i.e.* IQR) where whiskers denote the maximal and minimal values of the distribution. Values in Fig. 3e are represented as histogram distributions and mean ± SEM. Values in Fig. 4f are represented as mean ± SEM. In all representations, each point represents a measure obtained from a single animal. All statistical analysis was performed using MATLAB (MATHWORKS, Mountain View, CA, USA). Normality was tested Kolmogorov–Smirnov test. Asterisks denote the statistical significance calculated using Mann–Whitney U-test (Figs. 1b, 3c, d, e, 4b, c, f, 5a, b) and Chi-Square test (Fig. 4e). *p < 0.05; **p < 0.01, ***p < 0.001; ns, *p* > 0.05.

## Data Availability

The datasets analyzed in the current study are available from the corresponding authors upon request.

## References

[1] Boswell CW, Ciruna B (2017). Understanding idiopathic scoliosis: A New Zebrafish school of thought. Trends Genet..

[2] Bagnat M, Gray RS (2020). Development of a straight vertebrate body axis. Development.

[3] Grimme JD, Castillo M (2007). Congenital anomalies of the spine. Neuroimaging Clin. N. Am..

[4] Taniguchi Y (1976). Predictive physical manifestation for progression of scoliosis in Marfan syndrome. Spine.

[5] Abousamara O (2017). Scoliosis in down’s syndrome. J. Pediatr. Orthop..

[6] Huang TJ, Lubicky JP, Hammerberg KW (1994). Scoliosis in Rett syndrome. Orthop. Rev..

[7] Cheng JC (2015). Adolescent idiopathic scoliosis. Nat. Rev. Dis. Prim..

[8] Sebaaly A, Farjallah S, Kharrat K, Kreichati G, Daher M (2022). Scheuermann’s kyphosis: Update on pathophysiology and surgical treatment. EFORT Open Rev..

[9] Sardar ZM, Ames RJ, Lenke L (2019). Scheuermann’s Kyphosis: Diagnosis, management, and selecting fusion levels. J. Am. Acad. Orthop. Surg..

[10] Sørensen K (1964). Scheuermann’s Juvenile Kyphosis: Clinical Appearances, Radiography, Aetiology, and Prognosis.

[11] Faubel R, Westendorf C, Bodenschatz E, Eichele G (2016). Cilia-based flow network in the brain ventricles. Science.

[12] Olstad EW (2019). Ciliary beating compartmentalizes cerebrospinal fluid flow in the brain and regulates ventricular development. Curr. Biol..

[13] Thouvenin O (2020). Origin and role of the cerebrospinal fluid bidirectional flow in the central canal. Elife.

[14] Meiniel O (2008). The lengthening of a giant protein: When, how, and why?. J. Mol. Evol..

[15] Cantaut-Belarif Y, Sternberg JR, Thouvenin O, Wyart C, Bardet PL (2018). The Reissner fiber in the cerebrospinal fluid controls morphogenesis of the body axis. Curr. Biol..

[16] Grimes DT (2016). Zebrafish models of idiopathic scoliosis link cerebrospinal fluid flow defects to spine curvature. Science.

[17] Troutwine B (2019). The reissner fiber is highly dynamic in vivo and controls morphogenesis of the spine. Curr. Biol..

[18] Rose CD (2020). SCO-spondin defects and neuroinflammation are conserved mechanisms driving spinal deformity across genetic models of idiopathic scoliosis. Curr. Biol..

[19] Lu H, Shagirova A, Goggi JL, Yeo HL, Roy S (2020). Reissner fibre-induced urotensin signalling from cerebrospinal fluid-contacting neurons prevents scoliosis of the vertebrate spine. Biol. Open.

[20] Djenoune L (2014). Investigation of spinal cerebrospinal fluid-contacting neurons expressing PKD2L1: Evidence for a conserved system from fish to primates. Front. Neuroanat..

[21] Böhm UL (2016). CSF-contacting neurons regulate locomotion by relaying mechanical stimuli to spinal circuits. Nat. Commun..

[22] Sternberg JR (2018). Pkd2l1 is required for mechanoception in cerebrospinal fluid-contacting neurons and maintenance of spine curvature. Nat. Commun..

[23] Orts-Del’Immagine A (2020). Sensory neurons contacting the cerebrospinal fluid require the reissner fiber to detect spinal curvature in vivo. Curr. Biol..

[24] Stevenson NL (2017). Giantin-knockout models reveal a feedback loop between Golgi function and glycosyltransferase expression. J. Cell Sci..

[25] de Vos IJHM (2020). The novel zebrafish model pretzel demonstrates a central role for SH3PXD2B in defective collagen remodelling and fibrosis in Frank-Ter Haar syndrome. Biol. Open.

[26] de Vos IJHM (2018). Functional analysis of a hypomorphic allele shows that MMP14 catalytic activity is the prime determinant of the Winchester syndrome phenotype. Hum. Mol. Genet..

[27] McKay SD, Al-Omari A, Tomlinson LA, Dormans JP (2012). Review of cervical spine anomalies in genetic syndromes. Spine.

[28] Morin-Kensicki EM, Melancon E, Eisen JS (2002). Segmental relationship between somites and vertebral column in zebrafish. Development.

[29] Gistelinck C (2018). Zebrafish type I collagen mutants faithfully recapitulate human type I collagenopathies. Proc. Natl. Acad. Sci. U. S. A..

[30] Sun X (2020). Dstyk mutation leads to congenital scoliosis-like vertebral malformations in zebrafish via dysregulated mTORC1/TFEB pathway. Nat. Commun..

[31] Gray RS (2021). Postembryonic screen for mutations affecting spine development in zebrafish. Dev. Biol..

[32] Cantaut-Belarif Y (2020). Adrenergic activation modulates the signal from the reissner fiber to cerebrospinal fluid-contacting neurons during development. Elife.

[33] Quan FB (2015). Comparative distribution and in vitro activities of the urotensin II-related peptides URP1 and URP2 in zebrafish: Evidence for their colocalization in spinal cerebrospinal fluid-contacting neurons. PLoS ONE.

[34] Scheuermann H (1920). Kyfosis dorsalis juvenilis. Ugesk Leager.

[35] Tribus CB (1998). Scheuermann’s kyphosis in adolescents and adults: Diagnosis and management. J. Am. Acad. Orthop. Surg..

[36] Cahill PJ (2015). Sagittal spinopelvic parameters in scheuermann’s kyphosis: A preliminary study. Spine Deform..

[37] Bezalel T, Carmeli E, Kalichman L (2020). Introduction of the novel radiographic line (L5-kyphosis apex line) intended to evaluate scheuermann’s disease and postural kyphosis progression on standard lateral X-rays. Asian Spine J..

[38] Diebo BG, Varghese JJ, Lafage R, Schwab FJ, Lafage V (2015). Sagittal alignment of the spine: What do you need to know?. Clin. Neurol. Neurosurg..

[39] Le Huec JC, Thompson W, Mohsinaly Y, Barrey C, Faundez A (2019). Sagittal balance of the spine. Eur. Spine J..

[40] Marie-Hardy L, Khalifé M, Slimani L, Pascal-Moussellard H (2019). Computed tomography method for characterising the zebrafish spine. Rev. Chir. Orthop. Traumatol..

[41] Yamauchi Y, Yamaguchi T, Asaka Y (1988). Prediction of curve progression in idiopathic scoliosis based on initial roentgenograms. A proposal of an equation. Spine.

[42] Kohno S (2011). Factors predicting progression in early degenerative lumbar scoliosis. J. Orthop. Surg. (Hong Kong).

[43] Abelin-Genevois K (2021). Sagittal balance of the spine. Orthop. Traumatol. Surg. Res..

[44] Wenger DR, Frick SL (1999). Scheuermann kyphosis. Spine.

[45] Lowe TG (1999). Scheuermann’s disease. Orthop. Clin. North Am..

[46] Damborg F (2011). Genetic epidemiology of Scheuermann’s disease: Heritability and prevalence over a 50-year period. Acta Orthop..

[47] Urrutia J (2019). Scheuermann’s disease in patients 15–40 years old: A study to determine its prevalence and its relationship with age and sex using chest radiographs as screening tool. J. Orthop. Sci..

[48] Bezalel T, Carmeli E, Kalichman L (2019). Scheuermann’s disease: Radiographic pathomorphology and association with clinical features. Asian Spine J..

[49] Gerhard GS (2002). Life spans and senescent phenotypes in two strains of Zebrafish (Danio rerio). Exp. Gerontol..

[50] Hayes AJ (2013). Spinal deformity in aged zebrafish is accompanied by degenerative changes to their vertebrae that resemble osteoarthritis. PLoS ONE.

[51] Bearce EA (2022). Urotensin II-related peptides, Urp1 and Urp2, control zebrafish spine morphology. Elife.

[52] Gaillard A (2023). Urp1 and Urp2 act redundantly to maintain spine shape in zebra fi sh larvae. Dev. Biol..

[53] Lonner BS (2017). MRI screening in operative scheuermann kyphosis: Is it necessary?. Spine Deform..

[54] Demiroz S (2018). Intraspinal anomalies in individuals with Scheuermann’s kyphosis: Is the routine use of magnetic resonance imaging necessary for preoperative evaluation?. Asian Spine J..

[55] Abul-Kasim K, Schlenzka D, Selariu E, Ohlin A (2012). Spinal epidural lipomatosis: A common imaging feature in scheuermann disease. J. Spinal Disord. Tech..

[56] Yasuda T (2018). Clinical and imaging characteristics in patients undergoing surgery for lumbar epidural lipomatosis. BMC Musculoskelet. Disord..

[57] Halal F, Gledhill RB, Fraser FC (1978). Dominant inheritance of Scheuermann’s juvenile kyphosis. Am. J. Dis. Child..

[58] Fernandes LM (2020). Single-cell RNA-seq identifies unique transcriptional landscapes of human nucleus pulposus and annulus fibrosus cells. Sci. Rep..

[59] Löppönen T (2004). Childhood-onset osteoarthritis, tall stature, and sensorineural hearing loss associated with Arg 75-Cys mutation in procollagen type II gene (COL2A1). Arthritis Care Res..

[60] Karppinen J (2003). Radiologic phenotypes in lumbar MR imaging for a gene defect in the COL9A3 gene of type IX collagen. Radiology.

[61] Gao B (2022). Discovery and application of postnatal nucleus pulposus progenitors essential for intervertebral disc homeostasis and degeneration. Adv. Sci..

[62] Xie H (2023). Ependymal polarity defects coupled with disorganized ciliary beating drive abnormal cerebrospinal fluid flow and spine curvature in zebrafish. PLoS Biol..

[63] Didier R, Dastugue B, Meniel A (1995). The secretory material of the subcommissural organ of the chick embryo. Characterization of a specific polypeptide by two-dimensional electrophoresis. Int. J. Dev. Biol..

[64] Schindelin J (2012). Fiji: An open-source platform for biological-image analysis. Nat. Methods.

